# Impact of robotic surgery on systemic immune-inflammation index in gastric cancer patients: a retrospective cohort study

**DOI:** 10.1590/1806-9282.20250258

**Published:** 2025-08-08

**Authors:** Yiğit Düzköylü, Pınar Gülcan, Hüsnü Ozan Şevik, Oğuzhan Tekin, Hürü Ceren Gökduman, Erdal Karaköse, Sercan Yüksel, Zafer Teke

**Affiliations:** 1 Başakşehir Çam and Sakura City Hospital, Department of Gastrointestinal Surgery – İstanbul, Turkey.; 2 Başakşehir Çam and Sakura City Hospital, Department of General Surgery – İstanbul, Turkey.; 3 Başakşehir Çam and Sakura City Hospital, Department of Anesthesiology – İstanbul, Turkey.

**Keywords:** Gastric cancer, Inflammation, Systemic inflammatory response syndrome, Minimally invasive surgical procedures, Robotic surgical procedures

## Abstract

**OBJECTIVE:**

Robotic surgery has been gaining attention because of the physical and metabolic morbidity of the conventional open technique. The systemic immune-inflammation index has emerged as a recent and more reliable biomarker. In our single-center retrospective cohort study, we investigate systemic immune-inflammation index in robotic gastrectomy in order to show the advantageous effect on the immune system, which we think is the first study in the literature.

**METHODS:**

The study involved patients from a high-volume center for 32 months. The patients were allocated into three groups: patients with robotic (1), laparoscopic (2), and open surgery (3). Venous blood was derived on the postoperative 24th hour. The systemic immune-inflammation index scores were compared in three groups in terms of Group 1 vs. Groups 2 and 3, Groups 1 and 2 vs. Group 3, and compared with platelet-lymphocyte ratio, neutrophil–lymphocyte ratio scores.

**RESULTS:**

Robotic surgery was performed in 55 patients, laparoscopic surgery in 13 patients, and open surgery in 248 patients. In the comparison of minimally invasive surgery (Groups 1+2) vs. open surgery (Group 3), systemic immune-inflammation index, platelet-lymphocyte ratio, and neutrophil–lymphocyte ratio were found to be significantly lower in the minimally invasive surgery group (p<0.05). The comparison between robotic surgery patients (Group 1) and patients with laparoscopy/open surgery (groups 2 and 3) showed that systemic immune-inflammation index, platelet-lymphocyte ratio, and neutrophil–lymphocyte ratio were significantly lower in Group 1 (p<0.05). neutrophil–lymphocyte ratio, platelet-lymphocyte ratio, and systemic immune-inflammation index values were analyzed by receiver operating characteristic curve test, and the area under curve value of systemic immune-inflammation index was found to be higher in minimally invasive surgery group (robotic+laparoscopic) (p<0.05).

**CONCLUSION:**

The study reveals the superiority of robotic gastrectomy as a more feasible and high-quality procedure over conventional techniques in terms of better preservation of immune system function using a reliable and noninvasive serum biomarker.

## INTRODUCTION

Gastric cancer (GC) persists as a significant global public health concern, ranking as the fifth most prevalent cancer worldwide and the fourth leading cause of cancer-related mortality^1^. The gold standard curative treatment of gastric tumors is total surgical resection plus en-bloc lymphadenectomy with adjuvant or neoadjuvant therapy^2^. Despite the continued preference for conventional open gastrectomy, there has been a growing acceptance of minimally invasive surgical approaches due to the physical and metabolic morbidity associated with the procedure. Minimally invasive surgery (MIS) offers several advantages over traditional open surgery, including reduced postoperative pain, shorter hospital stays, and a lower risk of wound infection. Additionally, MIS is a safer and more cost-effective surgical approach, with a more favorable recovery course^3,4^. In the early 2000s, the advent of robotic surgery in the treatment of GC provided a novel and more comprehensive perspective, with published evidence indicating the superiority of the procedure in comparison to laparoscopy^5^.

In recent years, there has been a growing interest in comparing the outcomes of different surgical techniques, with a particular focus on the immune response of the patient and its effect on both recovery and survival rates. It is well-established that surgical procedures inevitably result in trauma to the patient, which in turn elicits a host immune response. This initiates a cascade of proinflammatory reactions, culminating in the synthesis of anti-inflammatory cytokines^6^. An uncontrolled response in either state may result in a systemic inflammatory response syndrome (SIRS), multiorgan failure, and even death^6,7^. The alterations in host immune response are also directly related to the tumor microenvironment, its development, and metastasis by promoting angiogenesis and cell proliferation^8^. The status of the current systemic immune inflammation can be demonstrated with serum-based biomarkers such as neutrophil (NE), lymphocyte (LY), and platelet (PLT) counts^9^. The neutrophil–lymphocyte ratio (NLR) and platelet–lymphocyte ratio (PLT) have been the subject of investigation as potential biomarkers in the diagnosis, prognosis, and surgical outcome of various tumors^10^. The systemic immune-inflammation index (SII) has recently emerged as a promising biomarker, combining these three cell counts in various tumor types to provide more consistent information, thereby facilitating a clearer understanding of the immune response^11^. The role of SII and its superiority to previous markers have been demonstrated in the diagnosis and prognosis of GC^10,12^.

In this single-center retrospective cohort study conducted at a high-volume center, we investigate the postoperative SII in robotic GC surgery to demonstrate the beneficial impact of MIS on the immune system. To the best of our knowledge, this is the first study of its kind in the literature.

## METHODS

This was a single-center, retrospective cohort study conducted in accordance with the principles of the Declaration of Helsinki. Ethical approval was obtained from the Clinical Research Ethics Committee of Istanbul Basaksehir Cam and Sakura City Hospital on 04/12/2024 with the registration number (2024-52). We strictly adhered to the Strobe guideline throughout the study.

Patients who underwent robotic gastrectomy between January 2021 and September 2024 were included in the study. The primary outcome was to ascertain the beneficial effects of robotic surgery in GC patients in terms of immune response. The secondary outcome was to examine the reliability of SII as a serum biomarker in comparison to previous studies on the diagnosis and postoperative follow-up of GC.

In order to be included in the study, participants had to have undergone curative surgical treatment for histopathologically confirmed gastric adenocarcinoma at our clinic during the specified time period, with verified serum biomarkers in the early postoperative course. Patients with a diagnosed hematological malignancy or immune system disorder, those who had previously undergone intra-abdominal surgery, those who had received a blood transfusion within 1 month prior to surgery, those who had been taking anticoagulant drugs for a long time, and those with a history of other systemic malignancies were excluded from the study. The exclusion criteria also included patients with a histopathologically confirmed diagnosis of tumors other than adenocarcinoma, procedures involving splenectomy, patients with an American Society of Anesthesiologists Physical Status (ASA_PS) classification of 3 or above, and patients who underwent conversion surgery (i.e., a change from MIS to open surgery).

The participants were divided into three groups according to the surgical approach employed: robotic, laparoscopic, or conventional open surgery. All three groups underwent the same surgical procedure including D2 lymphadenectomy. The decision regarding the type of procedure was based on the patient's consent, their body mass index, and the recommendations of the anesthesiologist. Robotic surgical procedures were conducted using the Da Vinci Xi system (Intuitive Surgical, Sunnyvale, CA, USA).

Venous blood of 2 mL was derived routinely from all patients on the postoperative 24th hour. The SII was calculated by the formula [SII=(platelet count x neutrophil count)/lymphocyte count]. Outcome measures included platelet (PLT, %), neutrophil (NEU, 10^9^/L), and lymphocyte (LYM, %) counts. The calculated SII scores were compared in three groups in terms of Group 1 vs. Groups 2 and 3 (robotic surgery vs. others), Groups 1 and 2 vs. Group 3 (MIS vs. open surgery). The SII score was also compared with platelet-lymphocyte ratio (PLR) and NLR scores.

### Statistical analysis

The statistical analysis of the data was conducted using the Statistical Package for the Social Sciences (SPSS) 25.0 software package. Categorical data were expressed as numbers and percentages, while continuous data were summarized as means, standard deviations, medians, minimums, and maximums, where appropriate. A chi-square test was employed for the comparison of categorical variables. In order to ascertain whether the parameters included in the study exhibited a normal distribution, the Shapiro-Wilk test was employed. In the absence of a normal distribution, the Mann-Whitney U test was employed for the analysis of paired group data. In all tests, the statistical significance level was set at p<0.05.

## RESULTS

**Figure 1:**
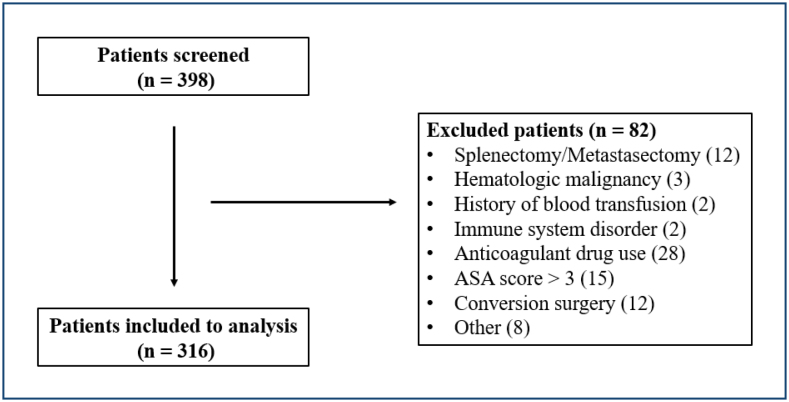
Flow-chart of patient selection.

A total of 316 patients were involved in the study (Figure 1), 222 of them males (70.3%) and 94 were females (29.7%), with a mean age of 64.3±11.1 (Med: 65). Robotic surgery had been performed in 55 patients (17.4%, Group 1), laparoscopic surgery had been performed in 13 (4.1%, Group 2), while open surgery had been preferred in 248 (78.5%, Group 3) patients (Table 1). The duration of hospital stay was shorter in the minimally invasive group. Similarly, postoperative complications were not found to be significantly different between groups in terms of Clavien-Dindo scale. Neoadjuvant chemotherapy had been preferred in 121 (38.3%) of the patients but was not found to be significantly related to inflammatory biomarkers.

**Table 1:** Demographics, tumor stage, serum biomarkers.

		Patients (n)	Percentage (%)
**Gender**			
	Male	222	70.3
	Female	94	29.7
**Operation type**			
	Robotic	55	17.4
	Laparoscopic	13	4.1
	Open	248	78.5
**T**			
	1	9	2.8
	1a	20	6.3
	1b	28	8.9
	2	28	8.9
	3	100	31.6
	4	3	0.9
	4a	119	37.7
	4b	9	2.8
**N**			
	0	87	27.5
	1	59	18.7
	1a	4	1.3
	2	48	15.2
	3	1	0.3
	3a	62	19.6
	3b	55	17.4
**M**			
	0	291	92.1
	1	25	7.9
		Mean±SD	Med (Min–Max)
Age		64.3±11.1	65 (29–87)
PLT		252.0±88.2	246.5 (59–662)
NEU		12.0±4.8	11.5 (2.28–28.2)
LYM		0.90±0.5	0.80 (0.12–3.55)
PLR		352.2±219.1	310.5 (17.8–1,772)
NLR		17.2±11.6	14.1 (1.29–92.3)
SII		4,382.5±3,544.5	3,408.8 (76.2–27,519.2)

SD: standard deviation; PLT: platelet; NEU: neutrophil; LYM: lymphocyte; NLR: neutrophil–lymphocyte ratio; SII: immune-inflammation index; PLR: platelet-lymphocye ratio.

In the comparison of MIS (Groups 1+2) vs. open surgery (Group 3), SII, PLR, and NLR were found to be significantly lower in the MIS group (p=0.001; p=0.001; p=0.021, respectively). There were no significant differences among other parameters (Table 2).

**Table 2:** Comparison of minimally invasive surgery and open surgery groups.

		Robotic & laparoscopic (n=68)	Open (n=248)	p-value
		n (%)	n (%)	
**Gender**				
	Male	45 (66.2)	177 (71.4)	0.406
	Female	23 (33.8)	71 (28.6)	
**T**				
	1	4 (5.9)	5 (2)	
	1a	5 (7.4)	15 (6)	
	1b	10 (14.7)	18 (7.3)	
	2	11 (16.2)	17 (6.9)	
	3	19 (27.9)	81 (32.7)	
	4	–	3 (1.2)	
	4a	19 (27.9)	100 (40.3)	
	4b	–	9 (3.6)	
**N**				
	0	18 (26.5)	69 (27.8)	
	1	23 (33.8)	36 (14.5)	
	1a	1 (1.5)	3 (1.2)	
	2	10 (14.7)	38 (15.3)	
	3	–	1 (0.4)	
	3a	10 (14.7)	52 (21)	
	3b	6 (8.8)	49 (19.8)	
**M**				
	0	65 (95.6)	226 (91.1)	0.227
	1	3 (4.4)	22 (8.9)	
		Mean±SD (Med)	Mean±SD (Med)	p-value
Age		62.2±9.3 63.5)	64.9±11.5 (66)	0.030
PLR		273.8±130.1 (251.9)	373.7±233.4 (331.9)	**0.001** **
NLR		14.6±9.7 (11.3)	17.9±12.0 (14.8)	**0.021** *
SII		3,222.3±2,478.9 (2,498.6)	4,700.6±3,726.2 (3,731.6)	**0.001** **

* p<0.05,

** p<0.01, Chi-square, Mann-Whitney U. SD: standard deviation; NLR: neutrophil–lymphocyte ratio; PLR: platelet-lymphocye ratio; SII: immune-inflammation index; T: extent of the tumor; N: extent of spread to the lymph nodes; M: presence of metastasis. Numerical values in bold are the parameters with statistically significant differences.

In order to find a possible advantage of robotic surgery in terms of determined serum biomarkers, the comparison between robotic surgery patients (Group 1) and patients with laparoscopy/open surgery (Groups 2 and 3) showed that SII, PLR, and NLR were significantly lower in Group 1 (p<0.001; p<0.001; p=0.023, respectively). There were no significant differences among other parameters (Table 3).

**Table 3:** Comparison of robotic surgery and laparoscopic/open surgery groups.

		Robotic (n=55)	Laparoscopic& open (n=261)	p-value
		n (%)	n (%)	
**Gender**				
	Male	40 (72.7)	182 (69.7)	0.659
	Female	15 (27.3)	79 (30.3)	
**T**				
	1	3 (5.5)	6 (2.3)	
	1a	3 (5.5)	17 (6.5)	
	1b	7 (12.7)	21 (8)	
	2	8 (14.5)	20 (7.7)	
	3	17 (30.9)	83 (31.8)	
	4	–	3 (1.1)	
	4a	17 (30.9)	102 (39.1)	
	4b	–	9 (3.4)	
**N**				
	0	14 (25.5)	73 (28)	
	1	17 (30.9)	42 (16.1)	
	1a	1 (1.8)	3 (1.1)	
	2	8 (14.5)	40 (15.3)	
	3	–	1 (0.4)	
	3a	9 (16.4)	53 (20.3)	
	3b	6 (10.9)	49 (18.8)	
**M**				
	0	52 (94.5)	239 (91.6)	0.458
	1	3 (5.5)	22 (8.4)	
		Mean±SD (Med)	Mean±SD (Med)	p-value
Age		63.4±8.9 (65)	64.5±11.5 (66)	0.284
PLR		262.7±135.1 (225)	371.1±228.8 (330.2)	**<0.001** **
NLR		14.4±9.9 (10.8)	17.8±11.9 (14.5)	**0.023** *
SII		3,071.0±2,537.6 (2,247.4)	4,658.8±3,666.5 (3,731.5)	**<0.001** **

* p<0.05,

** p<0.01, Chi-square, Mann-Whitney U. SD: standard deviation; NLR: neutrophil–lymphocyte ratio; PLR: platelet-lymphocye ratio; SII: immune-inflammation index. T: extent of the tumor; N: extent of spread to the lymph nodes; M: presence of metastasis. Numerical values in bold are the parameters with statistically significant differences.

NLR, PLR, and SII values were analyzed by the receiver operating characteristic (Roc) curve test to find a threshold value for serum biomarkers measured in the postoperative period. According to the analysis, an NLR of 15.80 and below was associated with an area under curve (AUC) of 59.1%, a sensitivity of 72.06%, and a specificity of 47.98% (p=0.018); a PLR of 240.3 and below was associated with an AUC of 63.3%, a sensitivity of 50%, and a specificity of 70.97% (p=0.002); SII value of 3,552.5 and below with a 63.9% AUC, 72.06% sensitivity, and 52.82% specificity value (p=0.002) among operation types. The AUC value of SII was found to be higher in the MIS group (robotic+laparoscopic) (p<0.05).

## DISCUSSION

The objective of the present study was to demonstrate the beneficial effects of robotic surgery for GC in terms of immunological system function, with a particular focus on SII, which has been demonstrated to be a reliable parameter for different stages of the cancer process in recent years. A database from a high-volume surgical center with extensive experience in GC surgery was subjected to evaluation.

GC remains a significant public health concern, with surgery remaining the primary curative treatment modality. In recent years, technological advances have led to a shift in technical preference from conventional open surgery to MIS, due to the considerable morbidity associated with the former^13^. Kitano et al. were the first surgeons to perform laparoscopic gastrectomy (LG) in 1994, and the technique subsequently gained widespread acceptance globally until recently^14^. Notwithstanding the reservations that have been expressed, a substantial body of evidence from studies and meta-analyses has demonstrated that the oncological outcomes are comparable between laparoscopic and open surgery, with an adequate number of dissected lymph nodes and a similar survival rate^4,15^. Approximately two decades after the initial LG, Hashizume et al. conducted the inaugural robotic gastrectomy (RG)^16^. As time has passed, although LG has been used widely, its technical limitations, including a two-dimensional view, dependence on individual surgical experience, and uncomfortable positioning, have prompted surgeons to seek the advantages of robotic surgery. These advantages include high-definition imaging and better peroperative instrumentation, tremor prevention, an increased number of harvested lymph nodes, a shorter hospital stay despite a longer operation time, a better recovery period, improved overall short-term results, and a shorter learning curve^5,17^. Furthermore, studies have demonstrated a reduction in the incidence of pancreatic fistula and intra-abdominal infection^18^.

A review of the literature reveals that MIS, in addition to other advantages, has a less deleterious impact on the patient's immune response when compared to conventional surgery in GC. This is associated with a decreased systemic inflammatory response, which is known to be important for enhanced postoperative recovery^15^. Bohne et al. observed a reduction in proinflammatory parameters following laparoscopy when compared to open surgery in patients with colorectal cancer^19^. It is well-established that an enhanced immune system plays a significant role in protecting against tumor development and the risk of metastasis^20^. Neutrophils are responsible for secreting reactive oxygen species and vascular endothelial growth factor, which can contribute to metastasis^21^. This is similar to the effect of increased platelet levels^22^. Conversely, lymphocyte activity has been demonstrated to exert a protective influence with respect to tumor proliferation and migration^23^. As these markers can be quantified via routine blood tests, they are readily accessible and cost-effective, prompting researchers to publish numerous studies on their diagnostic, postoperative, and survival rates in various cancer types, including GC^10^.

The SII has recently been released and is thought to be a more reliable biomarker, combining these ratios in a single measure. In their retrospective study examining the role of SII in the diagnosis of GC, Zhang et al. observed considerably elevated values in the patient cohort when compared to the healthy population, even at early stages^10^. Furthermore, the researchers demonstrated that SII exhibited superior diagnostic efficacy when compared to NLR and PLR alone, as evidenced by the ROC curve analysis. Similarly, the AUC was found to be more significant in the MIS group in the present study. SII was demonstrated to be a crucial parameter not only in the diagnostic phase but also in the postoperative period. Aoyama et al. demonstrated that SII is an independent risk factor for GC patients, affecting postoperative complications^23^. Furthermore, the researchers observed that the incidence of peritoneal recurrence was higher in the SII-high group over the long term, a finding that aligns with the results of a previous study^24^. Furthermore, a superior response to chemotherapy was observed in SII-low groups^25^. These findings may serve as predictors for the relationship between SII and survival in GC patients. In 2024, Yang and Wu demonstrated a reduction in overall and disease-free survival rates among GC patients with elevated SII levels in their meta-analysis^11^.

In light of these developments in MIS techniques and the identification of the SII as a biomarker of the immune system in cancer patients, our objective was to synthesize these findings and investigate the potential superiority of robotic surgery in the postoperative course as a safer and more beneficial procedure.

To the best of our knowledge, this is the first study to posit that robotic surgery in GC results in diminished levels of SII postoperatively, appraising the index in a manner distinct from that of preceding studies pertaining to diagnosis and survival. However, it should be noted that the present study is not without limitations. In particular, the postoperative blood samples were collected at a single time point, and it may be beneficial to consider evaluations at multiple time points following the operation to gain a more comprehensive understanding of the subject matter. Additionally, the present study did not differentiate between total and subtotal gastrectomy procedures. Although ours is a tertiary clinic and a preferred reference center, the single-center design of the study may have caused bias in the results. Another possible bias is the fact that all of the patients had not been operated on by a single team, and minimally invasive procedures had been performed by relatively more experienced surgeons. A specific cutoff value for SII was not identified, in contrast to the findings of studies examining the diagnostic and predictive value of SII. It is recommended that prospective, multicenter studies be conducted to confirm the results of this study.

## CONCLUSION

The findings of our retrospective single-center study indicate that MIS, particularly RG, is a more feasible and efficacious procedure than the conventional technique. This is due to the fact that it allows for the more reliable and noninvasive measurement of serum biomarkers, which in turn permits the better preservation of immune system function. It is our hope that these findings will contribute to the wider application of robotic surgery in GC, with the ultimate goal of improving outcomes. However, further studies are required to confirm these findings.

## Data Availability

The datasets generated and/or analyzed during the current study are available from the corresponding author upon reasonable request.
